# Bilateral adrenal histoplasmosis incidentally detected on ^18^F-FDG PET/CT in an immunocompetent man with primary adrenal insufficiency: A case report

**DOI:** 10.22038/aojnmb.2026.92421.1683

**Published:** 2026

**Authors:** Nitin Gupta, Amit Rana

**Affiliations:** 1 Department of Nuclear Medicine, Dr Rajendra Prasad Government Medical College (RPGMC), Tanda, Kangra, Himachal Pradesh, India; 2 Department of Oncology, Dr Rajendra Prasad Government Medical College (RPGMC), Tanda, Kangra, Himachal Pradesh, India

**Keywords:** Adrenal histoplasmosis, ^18^F-FDG PET/CT, Adrenal insufficiency, Fungal infection

## Abstract

Adrenal histoplasmosis is an uncommon manifestation of Histoplasma capsulatum infection and may closely mimic malignancy or granulomatous diseases on imaging. We describe an immunocompetent 51 year old man with 5 months of constitutional symptoms who underwent ^18^F-FDG PET/CT for evaluation of occult disease. The scan revealed intensely FDG avid bilateral adrenal masses with necrotic components, with no other abnormal sites. Biochemical testing confirmed primary adrenal insufficiency. CT guided adrenal biopsy, targeted to the most FDG-avid viable component, demonstrated necrotizing granulomatous inflammation with intracellular yeast forms consistent with Histoplasma capsulatum. Antifungal therapy with itraconazole plus glucocorticoid replacement led to clinical improvement and interval regression of adrenal lesions on follow-up. This case highlights the value of ^18^F-FDG PET/CT for detecting unsuspected adrenal histoplasmosis, localizing optimal biopsy targets, and facilitating timely treatment even in immunocompetent patients presenting with nonspecific symptoms.

## Introduction

 Histoplasmosis is a systemic mycosis caused by the dimorphic fungus Histoplasma capsulatum. Although pulmonary infection is most common, disseminated disease can occur and may involve the adrenal glands, occasionally causing primary adrenal insufficiency (1, 2).

 Adrenal involvement is frequently bilateral and may present as incidentally detected adrenal masses or with constitutional symptoms; in a retrospective follow up series of 40 patients with adrenal histoplasmosis, non-specific symptoms were common and adrenal insufficiency occurred in a substantial proportion, emphasizing the need for hormonal evaluation in bilateral adrenal disease (3).

 On contrast-enhanced CT, adrenal histo-plasmosis often shows bilateral enlargement with central low attenuation/necrosis and peripheral enhancement; however, these findings overlap with tuberculosis, lymphoma, metastases, and other granulomatous infections (4-7).

^18^F-FDG PET/CT can help identify metabolically active lesions, evaluate whole-body disease distribution, and guide biopsy toward viable tissue rather than necrotic areas (8, 9). We report an immunocompetent man in whom adrenal histoplasmosis was incidentally detected on ^18^F-FDG PET/CT and confirmed on image guided biopsy, with concurrent primary adrenal insufficiency.

## Case presentation

 A 51 year old man with no known comorbidities presented with 5 months of progressive weight loss (~7 kg), anorexia, intermittent low-grade fever, and right shoulder pain. He was a chronic smoker (approximately 60 pack years) with chronic cough and exertional dyspnea. There was no known occupational or travel exposure suggestive of endemic fungal infection.

 Examination showed mild dehydration and postural hypotension, without hyper-pigmentation or lymphadenopathy. Baseline blood counts, liver/renal function tests and inflammatory markers were within normal limits. Chest CT showed emphysematous changes without pulmonary nodules, consolidation, or mediastinal lymphadenopathy.

**Figure 1:**
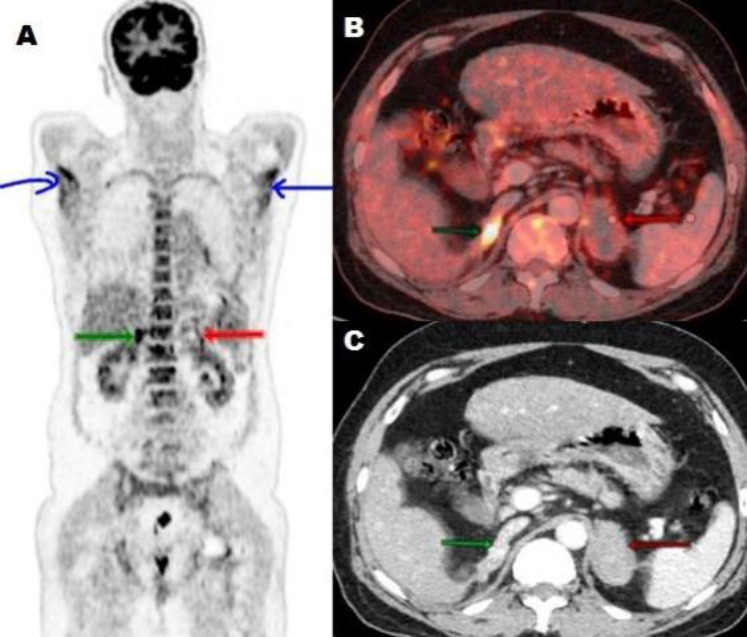
(**A**) Whole body ^18^F-FDG PET maximum intensity projection image shows increased uptake in the bilateral suprarenal regions (**arrows**) and in the shoulder/scapular regions. (**B**) Fused axial PET/CT and (**C**) corresponding contrast enhanced CT images show an FDG avid left adrenal lesion with central necrosis and an FDG avid enhancing right adrenal lesion

^18^F-FDG PET/CT was performed to evaluate for occult malignancy. Maximum intensity projection images showed FDG uptake in both suprarenal regions and in the scapular/ shoulder regions (Figure 1). 

**Figure 2:**
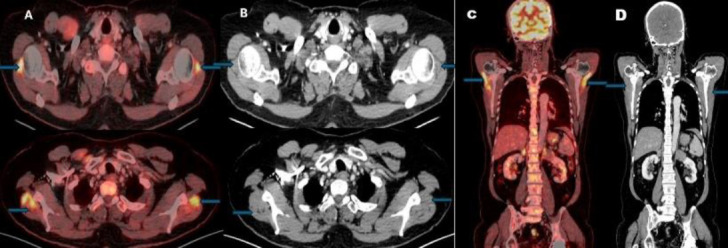
Fused PET/CT (**A**) and CT only (**B**) show increased FDG uptake at bilateral shoulder periarticular muscles in lateral aspect, and along supraspinatus/infraspinatus and teres muscles, without a focal mass or structural abnormality on CT (**blue arrows**). Coronal fused PET/CT images show diffuse increased FDG uptake along the scapular muscles (**C**) and corresponding CT images (**D**) (**blue arrows**) demonstrate no infrascapular soft tissue mass or typical alternating fat fibrous streaks helping exclude elastofibroma dorsi as the cause of FDG uptake

 On fused axial PET/CT and corresponding contrast enhanced CT images, the left adrenal gland was enlarged with a necrotic component and peripheral FDG uptake (SUV_max_=7.9), and the right adrenal gland showed an enhancing FDG avid mass. No other abnormal FDG uptake was identified. The shoulder region uptake localized to periarticular muscles around the humeral heads (including teres and supraspinatus/ infraspinatus regions) without focal CT abnormality. Transaxial as well as coronal images through the inferior scapular tips showed absence of the typical infrascapular soft tissue mass of elastofibroma dorsi (Figure 2).

 Morning serum cortisol was low (4.2 µg/dL; reference 6-23 µg/dL), and an ACTH stimulation test confirmed primary adrenal insufficiency. Anti-adrenal antibodies were negative.

**Figure 3:**
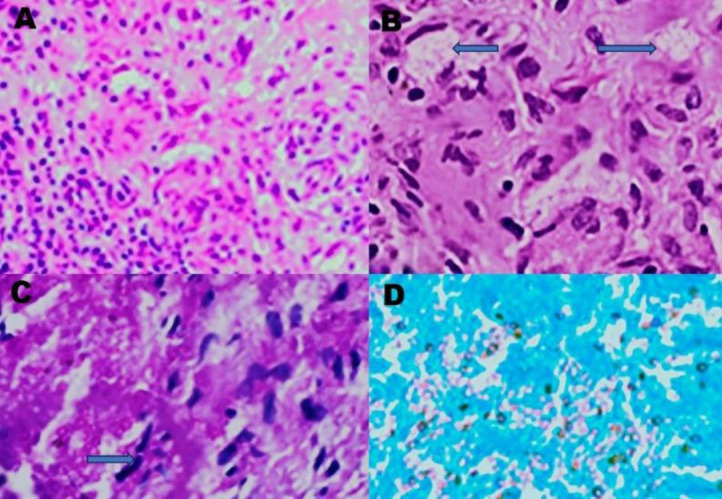
Photomicrographs of adrenal biopsy. (**A**, **B**) Necrotizing granulomatous inflammation with numerous intracellular yeast forms on H&E. (**C**) Periodic acid-Schiff and (**D**) Grocott methenamine silver stains highlight fungal elements consistent with Histoplasma capsulatum

 CT guided biopsy was directed to the most FDG avid viable component of the adrenal lesion. Histopathology showed necrotizing granulomatous inflammation with numerous 

intracellular yeast forms consistent with Histoplasma capsulatum; fungal elements were highlighted on periodic acid-Schiff and Grocott methenamine silver stains (Figure 3). Serum and urine Histoplasma antigen assays were positive.

 Oral itraconazole (200 mg twice daily) and hydrocortisone replacement were initiated. The patient showed clinical improvement and biochemical stabilization, with interval reduction in adrenal lesion size on follow up imaging at 6 months.

## Discussion

 Adrenal histoplasmosis is an important, potentially reversible cause of bilateral adrenal masses and may present in immunocompetent patients with nonspecific systemic symptoms. Because imaging appearances overlap with tuberculosis and malignancy, tissue diagnosis is often necessary, and adrenal function should be assessed in all patients with bilateral adrenal disease (3-7).

 CT features vary with disease phase. Acute disease may preserve adreniform contours, whereas subacute/chronic infection typically produces heterogeneous low attenuation from necrosis with peripheral enhancement; healing may be accompanied by calcification (4-7). 

 These patterns are not specific, and similar appearances can be seen with adrenal tuberculosis, lymphoma, metastases, and other fungal infections, reinforcing the role of biopsy (4-7).

^18^F-FDG PET/CT can be particularly useful in this setting. FDG uptake reflects inflammatory and granulomatous activity and can help select biopsy, potentially improving diagnostic yield compared with sampling necrotic cores. In addition, whole body PET/CT can exclude other metabolically active sites and provide a baseline for response assessment (8, 9).

 In our case, the additional finding of bilateral shoulder region FDG uptake prompted targeted evaluation. FDG uptake in the shoulder girdle may be caused by physiologic muscle activity, inflammatory tendinopathy/bursitis, degenerative (10).

 Elastofibroma dorsi is a benign fibroelastic pseudotumor typically located deep to the inferior scapular tip between the scapula and chest wall, frequently bilateral, and may demonstrate mild to moderate FDG uptake, leading to incidental detection on PET/CT (11-13). Characteristic CT features include a poorly defined soft tissue mass with alternating fibrous and fatty streaks in the infrascapular region (11). In our patient, FDG uptake localized to periarticular shoulder muscles without an infrascapular mass on short axis axial and also coronal images (Figure 2), making elasto fibroma dorsi unlikely; the pattern was more compatible with benign/inflammatory muscular uptake (10).

 Treatment of adrenal histoplasmosis generally follows disseminated histoplasmosis recommendations, with oral itraconazole for non-severe disease and amphotericin B (often liposomal) for severe disease, alongside glucocorticoid replacement when adrenal insufficiency is present (2). Even after radiologic regression, adrenal insufficiency may persist due to glandular destruction, and long term follow up is advised (3).

## Conclusion

^18^F-FDG PET/CT can reveal unsuspected adrenal histoplasmosis in immunocompetent patients with nonspecific systemic symptoms, assist in selecting optimal biopsy targets, and provide a baseline for treatment response. In bilateral adrenal disease, early hormonal evaluation and prompt tissue diagnosis enable timely antifungal therapy and appropriate steroid replacement, improving clinical outcomes.
